# Peripheral primitive neuroectodermal tumors: A retrospective analysis of 89 cases and literature review

**DOI:** 10.3892/ol.2020.11385

**Published:** 2020-02-12

**Authors:** Liming Gao, Yingying Zhu, Xiaohua Shi, Zhiqiang Gao, Xingming Chen

Oncol Lett 18: 6885-6890, 2019; DOI: 10.3892/ol.2019.11011

Subsequently to the publication of the above article, it has been noted that the article was erroneously classified as a ‘Review’ on the Journal's website and in Web of Science [as denoted by the inclusion of “(Review)” at the end of the title]. Although this retrospective analysis of 89 cases of peripheral primitive neuroectodermal tumors does include a review of the literature, the article, in itself, is not a review-type article. This error in the classification of the article type occurred during the pre-press stages.

All the authors agree to this Erratum, and the Editor apologizes to the authors for the inconvenience caused.

